# Impact of Using Facemasks on Literacy Learning: The Perception of Early Childhood Education Teachers

**DOI:** 10.3390/ejihpe12060048

**Published:** 2022-06-17

**Authors:** Diego Vergara, Álvaro Antón-Sancho, Juan-José Maldonado, María Nieto-Sobrino

**Affiliations:** 1 Department of Mechanical Engineering, Catholic University of Ávila, C/Canteros, s/n, 05005 Avila, Spain; 2 Department of Mathematics and Experimental Science, Catholic University of Ávila, C/Canteros, s/n, 05005 Avila, Spain; alvaro.anton@ucavila.es; 3 Department of Education, Catholic University of Ávila, C/Canteros, s/n, 05005 Avila, Spain; maldonadochicajuanjose@gmail.com (J.-J.M.); maria.nieto@ucavila.es (M.N.-S.)

**Keywords:** literacy, preschool, speech and hearing, therapeutic pedagogy, COVID-19 pandemic

## Abstract

In this work, quantitative research is carried out on the importance that educators give to literacy work in early childhood education classrooms and the impact that the COVID-19 pandemic and the use of facemasks have had on it. To this end, a survey designed for this purpose has been used, which has been passed on to a set of 112 Spanish early childhood educators. The teachers surveyed occupy different positions in the classroom (tutors, support technicians, specialists in bilingualism, therapeutic pedagogy and speech and hearing), and, in addition, they themselves learned to read from different methods of literacy learning (synthetic or analytical). The results found in this study indicate that educators express intermediate evaluations of the importance of literacy work in the classroom, higher if it is done through digital resources, and higher for the synthetic method than for the analytical method. In addition, the impact of the use of masks on literacy learning was rated as very negative. On the other hand, gaps have been identified in the above perceptions by the position occupied in the classroom and by the method used to learn to read. Finally, some actions are suggested to homogenize the perceptions of the different professionals, and some lines of research are proposed.

## 1. Introduction

The COVID-19 pandemic situation has been a turning point in daily life, resulting in major changes in daily, social, and work habits [1,2,3]. Among these transformations, the imposition of the mandatory use of facemasks in many countries as a sanitary measure stands out [4]. This fact has not left the educational field indifferent, which has been affected by the alteration of word and emotion recognition processes [5,6].

Language acquisition is a natural process that occurs in early childhood, which is directly related to the child’s socioemotional development [7]. It should be noted that, from about 2 years old, children undergo a language learning process, most of which takes place in the classroom [8]. However, after the pandemic situation, this learning process may have been truncated by the use of facemasks by teachers [5].

After a period based on online teaching, especially in the early stages of the pandemic, the return to schools has meant the adoption of safety and hygiene measures to prevent the spread of the virus [9], highlighting the mandatory use of facemasks in enclosed spaces. These precautions have been treated to different extents depending on the educational level or specialty, with the exclusion of teachers, who have not been able to do without them [10]. This fact has led to a new challenge for the teaching–learning process due to the sound attenuation caused by the use of the facemask by the early childhood education teachers, but also due to the lack of visibility of a large part of the interlocutor’s face [11].

This visual–gestural component is part of the basis for optimal communication between transmitter and receiver. In this sense, the sound affection caused using the facemask fluctuates according to its typology, oscillating for surgical facemasks around 4 dB, while for FFP2 around 12 dB [12]. In the same way, the loss of vision of part of the sender’s face and facial expressions affects the correct understanding of the message since nonverbal language is also affected [13].

**Figure 1:**
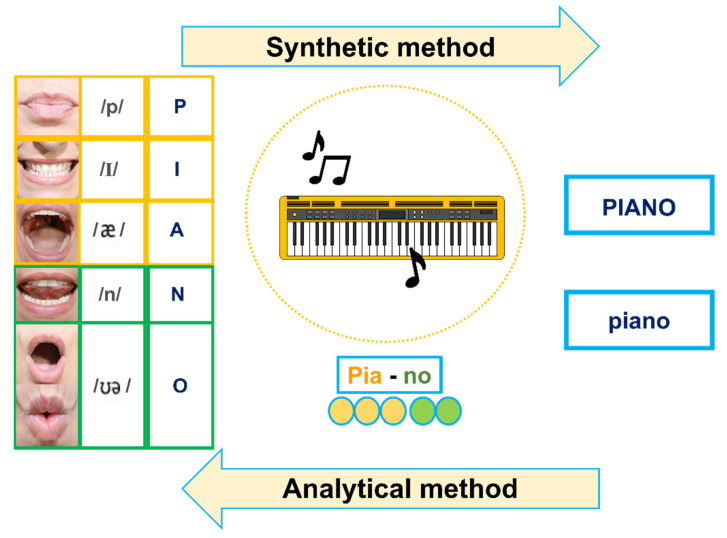
Scheme of both synthetic and analytical methods for literacy learning.

This attenuation of sound in the transmission of the message causes various effects in the sender, e.g., vocal and respiratory efforts that cause a state of fatigue in the subject [11,14]. This fact translates in the educational environment into a communication whose fundamental component is that of a state of anxiety, as well as a lack of understanding due to the use of shorter messages, and lack of information [14]. This, in turn, has repercussions on the correct development of literacy, understood as the process of learning to read and write, since knowledge of oral vocabulary directly improves students’ reading skills [15,16]. Literacy is an educational area that is usually worked on in early childhood education (educational stage from 3 to 6 years of age), through two main methods: synthetic and analytical (Figure 1). Synthetic is referred to the teaching that starts from the simplest to the most complex, i.e., begins with the study of phonemes (sounds) associated with letters and syllables, which allows for greater articulation of speech during the reading process and better decoding of words, especially improving the recognition of new words, which is a more effective method in the teaching of literacy. Conversely, analytical refers to the process by which children learn from more complex structures, that is, from the global (words and phrases) to the simplest structures (letters and syllables), improving the child’s autonomy in this process. This can be a challenge for children since, during the literacy learning process, they must distinguish the parts from the whole, which makes the process more complicated. Both approaches to literacy are affected by the visual and phonological impact of the use of facemasks [16,17].

In this regard, the use of transparent facemasks in the teaching-learning process seems to be a resource that solves part of the limitations in communication generated by opaque facemasks, since the visual signals of the communication process are exposed [18]. This favors the relationships established in the classroom, but also the learning of language from imitation and phoneme recognition at initial levels [5,19]. However, the sound affection is reduced in this type of facemasks, being up to 13.3 dB in those that have a window, and around 21 dB in those that are completely transparent [19].

Despite this, in noisy situations, as may be the case in a kindergarten classroom, the use of transparent facemasks is strongly recommended, since the sound attenuation is compensated by a better view of the facial field of the transmitter. This type of facemask in turn leads to access to components that allow the message to be deciphered, e.g., lip reading or the visualization of facial expressions that directly expose the emotional state of the subject [20]. This means breaking down the physical barrier that impedes the intelligibility of the message, providing the receiver with a state of confidence and peace of mind in view of the possibility of having more resources for greater understanding in the communication process [20]. On the other hand, although this type of transparent facemask has the disadvantage of being susceptible to generate a “fog” effect due to the respiratory process, this can be solved with both homemade and commercially available correctors for this purpose [5,20,21].

Even so, to counteract the sound affectation caused by facemasks, the educational world has often resorted to the use of microphones to improve communication [21], which has given better results—in general—with transparent facemasks, as they present a higher level of perception of the orofacial system [20]. However, it should be noted that for a correct understanding and cognitive processing of speech to occur, it is necessary to see the entire face of the sender without any physical barrier [22].

In addition to this circumstance, the use of the facemask by early childhood education students, who, despite not being obliged to use it—unlike teachers—have shown great frustration due to communication and socialization problems between student–student and student–teacher [23]. In this regard, another aggravating factor is the alteration of the correct pronunciation of phonemes, which will hinder the teaching work both at a general and specific level for the improvement of speech and language disorders [24], which occurs in speech and hearing (SH) or therapeutic pedagogy (TP) classrooms.

Since the beginning of the COVID-19 pandemic, there have been publications warning of the difficulties that the introduction of facemasks in schools posed for education, at the level of communication, interpretation of language and expression of emotions [25]. Some of these studies have focused on demonstrating the negative impact that the use of facemasks has at the academic level on the learning of some specific areas among adolescent students, such as physical education [26,27], although no adverse medical effects on children’s or adults’ breathing have been described, although respiratory discomfort has been reported [28]. However, several studies have analyzed the impact of using facemasks on the management and expression of emotions and affect in preschool and elementary school children and young people in secondary school.

In particular, it has been shown that facemasks have made it difficult for children to express feelings of disgust and have made them more prone to anxiety and fear [29], an effect that is particularly intense in children in early childhood education [30]. In adolescents, an increase in anxiety derived from the use of masks has been observed [31]. As for preschool, there are studies that explore the impact of the COVID-19 pandemic on early childhood education, but they are usually focused on describing the effects of the migration of education to virtual environments [32,33], or in exposing the experience teachers have had and the impact of the pandemic at the occupational level [34]. Other papers explore teachers’ and parents’ views on the overall impact of the pandemic, concluding that it has had a negative effect on the overall development of children in early childhood education, particularly in science, mathematics, and art [35]. However, as far as it has been possible to explore, there are no studies that analyze the impact that the use of masks has had, within the global phenomenon of the COVID-19 pandemic, on the concrete learning of reading and writing in early childhood education. Moreover, the perspective of early childhood teachers in this regard has also not been the subject of intense analysis in the preceding literature.

This way, this research is focused on the impact of the COVID-19 pandemic, particularly the use of facemasks, on literacy learning in early childhood education (3 to 6 years of age). Specifically, a statistical analysis is made of the opinion of early childhood education teachers in Spain regarding the use of facemasks in the classroom and the problems this generates in relation to the teaching–learning process of literacy at this educational stage. The study distinguishes the opinions of the different generalist and specialist teachers involved in the teaching of literacy and also differentiates by the method (synthetic or analytical) with which the participating teachers learned to read and write.

Specifically, the general objective of this research is to describe the opinion of early childhood education professionals on (i) literacy work in the classroom, (ii) the different methods for teaching it, and (iii) the influence of the COVID-19 pandemic and the use of facemasks on the development of literacy in students. In particular, the following specific objectives are pursued in this work: (i) to analyze the assessments of Spanish early childhood education teachers about the importance of working on literacy in the classroom and of doing so through digital resources; (ii) to study the assessments made by these professionals of the effectiveness of each of the possible methods of working on literacy (synthetic and analytical); (iii) to examine the influence of the use of facemasks on the achievement of literacy objectives in early childhood education during the COVID-19 pandemic; (iv) to identify differences in the above assessments by the teacher’s position in the classroom and, within each possible position, by the method with which the teacher learned to read; and (v) to analyze whether the COVID-19 pandemic has altered participants’ perception of the need for classroom reinforcement with a speech-language pathologist.

**Figure 2:**
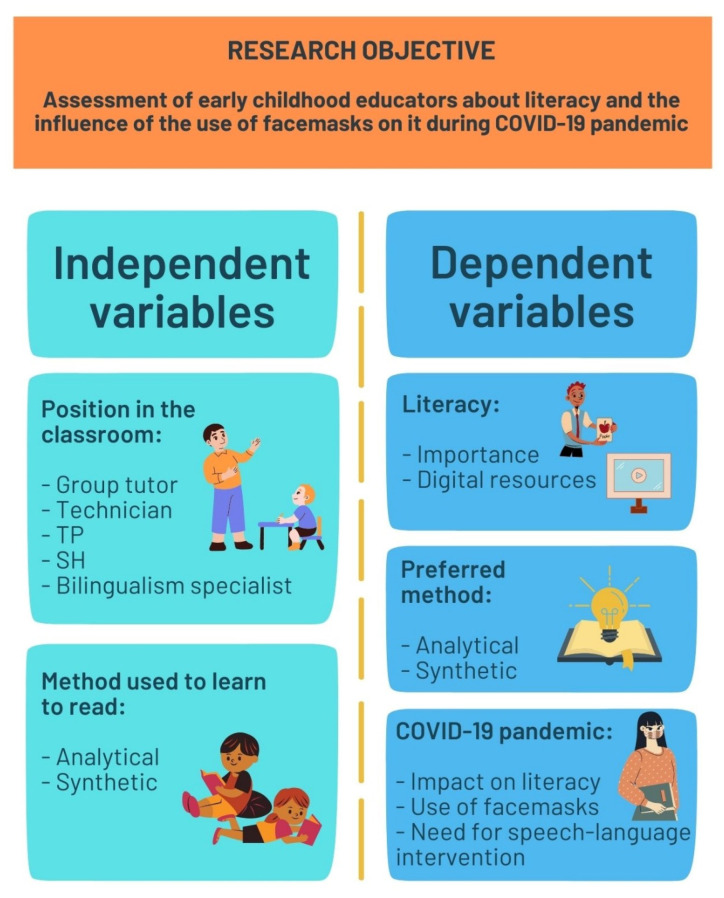
Research objective and variables.

Two independent variables are considered in the research (Figure 2): (i) position occupied in the classroom; and (ii) literacy learning method with which the participant learned to read. The position occupied in the classroom is the main independent variable. It is a polytomous variable whose values reach all possible early childhood education professionals working in the field of literacy: (i) teachers specializing in bilingualism; (ii) SH specialists; (iii) TP specialists; (iv) early childhood education teachers acting as group tutors; and (v) early childhood education technicians acting as support staff. The variable measuring the method by which participants learned to read is a dichotomous variable whose possible values are the analytical method or the synthetic method.

The following dependent variables are defined as quantitative variables measured on a scale of 1 to 5:

- Importance given by the participants to the teaching of literacy in early childhood education;
- Importance of the use of digital resources and information and communication technologies (ICT) in the process of teaching literacy in early childhood education;
- Assessment of the effectiveness of the analytical method in learning literacy;
- Assessment of the effectiveness of the synthetic method in learning literacy;
- Assessment of the impact of the COVID-19 pandemic on the development of literacy in early childhood education students;
- Degree of negative impact of the use of facemasks on the development of literacy in early childhood education students;
- Perception of the need for speech-language intervention in the classroom before and after the pandemic.

## 2. Materials and Methods

### 2.1. Participants

This study involves 112 active Spanish early childhood education professionals, including generalist teachers and technicians and also specialists in different educational fields related to literacy. Specifically, among the participants there are group tutors and support technicians, teachers specializing in bilingualism, and teachers specializing in SH (Teacher of the Speech and Hearing Handicapped, TSHH) or TP. Participants were chosen through a non-probability convenience sampling process.

**Figure 3:**
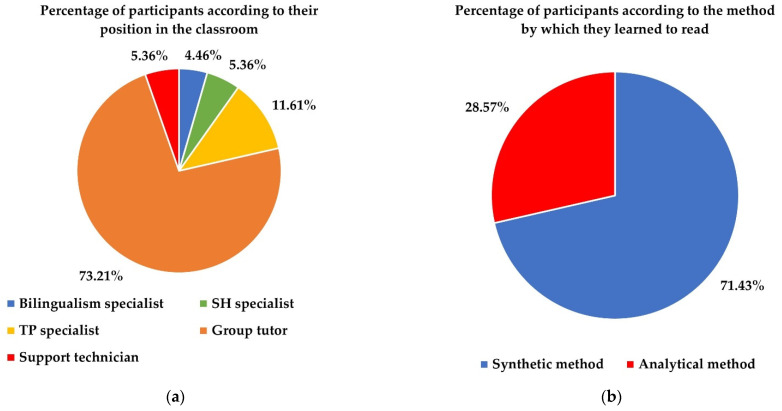
Distributions of participants by: (**a**) position held, and (**b**) method by which they learned to read.

The distribution of the proportions of each of the above positions in the sample is shown in Figure 3. As can be noted, there is a notable majority of teachers acting as group tutors. The distribution of participants according to the method by which they learned to read is also shown in Figure 3. Most of the participating teachers learned through the synthetic method. To conclude the description of the sociological profile of the participants, it is noteworthy that there is a large majority of females (91.07%) versus males (8.93%), the latter being only classroom tutors. Almost half of the participants are between 41 and 50 years old (46.43%), 33.04% are 40 years old or younger and 20.53% are over 50 years old. Only 6.25% are under 30 years old. Regarding teaching experience, 75.00% of the participants have more than 10 years of teaching experience, and 87.50% have more than 5 years of experience. Therefore, they have, in general, extensive teaching experience.

### 2.2. Instrument

In this research, a survey designed for this purpose has been used to delimit the sociological and professional profile of the participants and to obtain their opinions on the variables studied. In the survey, the following aspects of the sociological and educative profile of the participants are assessed: (i) gender; (ii) age; (iii) position held in the early childhood education classroom; (iv) teaching experience; and (v) method by which the participant learned to read (synthetic or analytical). Participants are then asked a set of 15 Likert-type questions valued from 1 to 5 to measure the teachers’ perception of the dependent variables analyzed (Table 1). In the answers to these questions, 1 means the lowest valuation and 5 is the highest valuation.

The validation of the instrument was carried out by means of a factor analysis, the computation of internal consistency parameters and an assessment of convergent validation by means of Pearson’s correlation coefficients. The answers of the first 13 questions of the survey (Table 1) were subjected to an EFA with Varimax rotation, which has determined the existence of six latent factors that explain this part of the survey (Table 2). The factor weights of questions 1, 4, 5, 10, and 13 are greater than 0.90, so the answers to these questions can be considered redundant and, consequently, have been removed from the analyzed results. The six-factor theoretical model derived from the EFA allows explaining the survey results with statistical significance (chi-square = 5.59, *p*-value = 0.78) and explain 64.3% of the variance (Table 3). The factors identified in this theoretical model correspond exactly to the first six dependent variables defined in the study. The last dependent variable (which measures the perception of the need for speech-language intervention in early childhood education before and after the pandemic) has been assessed by comparing the mean answers to questions 14 and 15 of the survey.

The statistics of the CFA endorse the theoretical model derived from the EFA. Indeed, the incremental fit indices are appropriate (AGFI = 0.8786; NFI = 0.8744; TLI = 0.9650; CFI = 0.9783; IFI = 0.9800) and the absolute fit indices are also good (GFI = 0.9375; RMSEA = 0.0403; AIC = 103.6463; chi-square/df = 1.1661). The validation of the psychometric dimensions of the survey has been tested based on Pearson’s correlation coefficients (Table 4). These correlations are weak between the different variables analyzed, but strong for each of the variables with respect to the survey. All the computed coefficients are statistically significant, with a significance level of 0.05.

### 2.3. Procedure

In this work, quantitative research is performed based on the answers to the survey that has been passed to the participating teachers. They were contacted by email and were given access to the survey instrument through GoogleForms^TM^. The teachers answered to the survey voluntarily, freely, and anonymously. The protocol was approved by the Ethics Committee of the research project to which the authors belong. All the answers were validated (an answer was considered valid when it was complete). The validation of the instrument was conducted after the response collection process before statistical analysis of results.

### 2.4. Data Analysis

The validation of the survey was carried out by means of an exploratory factor analysis (EFA) of the answers, which served to identify the latent factors that explain the survey. Questions 14 and 15 of the survey have not been introduced in the EFA because the intention is assessing the last dependent variable of the study (perception of the need for speech-language intervention) by comparing the mean results of question 14 (perception before the pandemic) and question 15 (perception after the pandemic). The theoretical model derived from the EFA was confirmed by means of confirmatory factor analysis (CFA) statistics. The validation of the psychometric dimensions was achieved through the computation of Pearson correlation coefficients of the different factors defined by the EFA among themselves and with respect to the global survey. The descriptive statistics of the answers of the first 13 questions of the survey (means and standard deviations) have then been analyzed, both globally and differentiating the participants according to their position in the classroom. The ANOVA test was applied to identify significant differences in the answers by position held. In the variables where such differences exist, the multifactor ANOVA test (MANOVA) has been applied to analyze whether, within each position, there are differences in the corresponding assessments due to the method by which the participants learned to read. Finally, the mean values of questions 14 and 15 were compared using the t-test and the ANOVA test (when the sample is differentiated by the position occupied in the classroom). In all tests, 0.05 has been taken as the level of statistical significance.

## 3. Results

In Table 5 it is shown that, in general, the survey respondents give an intermediate importance to literacy learning in early childhood education, but that they consider the synthetic method to be more efficient than the analytical method. Both in the assessment of literacy and in that of teaching methods, the deviations are high (above a quarter of the mean). On the other hand, the valuation of the use of digital resources for literacy learning is high and with a moderate deviation. The participants believe that the use of facemasks during the pandemic has had a very negative influence on the development of literacy in early childhood education students, with a low standard deviation. Participants’ assessment of the impact of the pandemic on the students’ literacy development during the pandemic is intermediate.

Differentiating the participants by the position they occupy in the classroom, significant differences are observed in the mean values of the importance of literacy in early childhood education and of the influence of the use of facemasks on the students’ literacy development (Table 6). SH and TP specialists are the ones who give the least importance to literacy work in the classroom, while teacher tutors and technicians give the highest valuations in this regard. Bilingualism teachers are the professionals who most intensely warn of the negative effects of facemasks on literacy.

**Figure 4:**
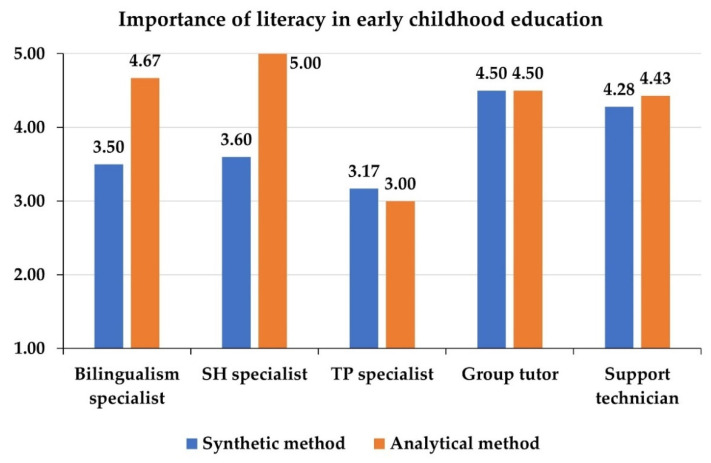
Mean values (out of 5) of the importance of literacy learning in early childhood education, differentiating by the position occupied in the classroom and the method by which the participants learned to read.

**Figure 5:**
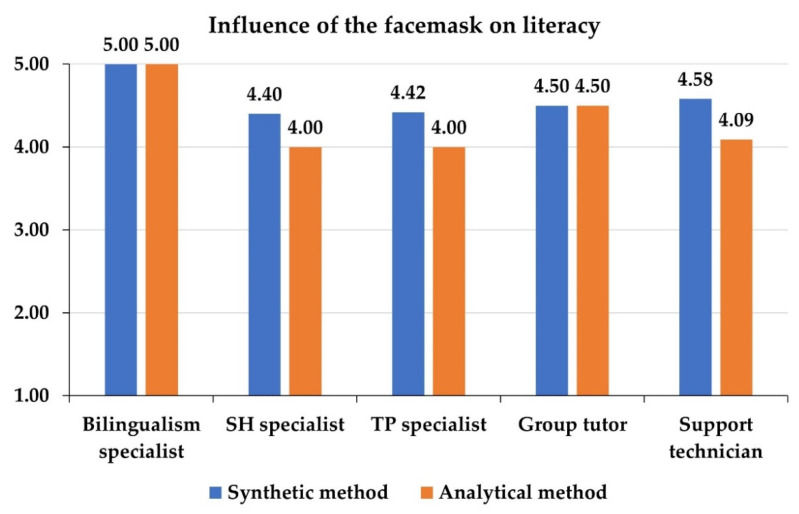
Mean values (out of 5) of the influence of the facemasks on literacy, differentiating by the position occupied in the classroom and the method by which the participants learned to read.

Next, the behavior of the participants’ opinions is analyzed for the two independent variables that show significant differences according to the teacher’s position in the classroom (importance of literacy and negative influence of the use of facemasks). Specifically, Figure 4 and Figure 5 show the mean values of the participants for each of these variables, differentiating by the method with which they themselves learned to read. As can be observed, in all positions, those who learned to read with the analytical method give a higher value to literacy than those who learned with the synthetic method, except among TP specialists (Figure 4). These differences are statistically significant (MANOVA’s F = 4.1434, *p*-value = 0.0039). Among group tutors, there is no difference in the evaluation of the importance of literacy when the sample is differentiated by the method used to learn to read. However, the participants who learned to read with the synthetic method are those who perceive a more negative influence of the use of the facemasks on the development of literacy in all positions and with significant differences (MANOVA’s F = 4.0928, *p*-value = 0.0459), except for the bilingualism specialists, among whom no differences by learning method are appreciated (Figure 5).

In response to questions 14 and 15 (Table 1), that value the perception of the need for speech-language intervention, participants assessed with a mean of 3.30 their need for such speech therapy in the early childhood classroom before the pandemic, and with a 3.80 their perception of it after the pandemic. The t-test statistics (t = −3.3731, *p*-value = 0.0009) found the difference between the means to be significant. Therefore, the pandemic has led to a significant increase in teachers’ assessment of the need for speech-language intervention in the early childhood classroom. In this regard, the ANOVA test does not identify statistically significant differences by position in the classroom (Table 7), so that the increase in mean perception is approximately homogeneous across all positions.

## 4. Discussion

Numerous studies have shown that the use of face masks during the COVID-19 pandemic has caused difficulties in children to adapt to them, problems of functional discomfort [10], compatibility with the use of glasses or breathing difficulties [26,36] and even psychological problems, such as anxiety or sadness [5,6,18,37,38]. Educators have warned about the negative influence that the use of facemasks has, in particular, on the proper development of children’s social life and their interaction with others [39,40], on the development of communication skills [13,21], on sound recognition and vocal self-perception [11,14,19,20], and on the development of speech skills [11,12,41]. This paper has analyzed the influence of the COVID-19 pandemic and, particularly, the use of facemasks, on the development of literacy objectives in early childhood education students, from the point of view of educators. In the analysis, emphasis has been placed on the identification of differences in the perceptions expressed due to the different specialties exercised by teachers in the early childhood education classroom.

The results show that, in general, educators give intermediate–high assessments to literacy learning in early childhood education, with these valuations being higher if digital resources are introduced (Table 5). In addition, the pandemic has led to a significant increase in educators’ assessment of the need for speech-language intervention in the classroom. This increase is approximately homogeneous across the different positions occupied by teachers in the classroom (Table 7). The results regarding the use of digital resources are in line with previous work that confirms the good learning results that this type of tool achieves in children in early childhood education when applied to the specific field of literacy [16,42]. It is surprising that the perception of the importance of working on literacy is not more favorable than the results suggest, given that the specialized literature supports the importance of this area for language development from early childhood [43,44]. The high standard deviation of the responses on the importance of literacy (Table 5) shows, however, the lack of unanimity in the assessments. This lack of consensus is, in turn, a consequence of the gap that exists in this regard among the different early childhood education professionals (Table 6). Specifically, specialists in bilingualism, SH and TP give lower ratings to literacy teaching than teachers and generalist technicians. These results show that group tutors and technicians are probably more familiar with the so-called Critical Age Hypothesis (CAH), which postulates the existence of a critical age range outside which language learning is more difficult and laborious [45]. Although different critical periods are identified according to the different language skills, in any case the CAH indicates that these periods are located before the age of six, both for the acquisition and learning of the mother tongue [46,47] and for second languages [48,49].

In any case, this study shows that generalist early childhood education teachers give greater importance than specialists to literacy work in the early childhood education stage (Table 5). This may be because specialist teachers have much more limited contact with children, which may mean that they do not perceive their needs in literacy as directly. All this suggests the need to increase specific training on literacy learning in the curricula of early childhood education professionals. In the case of bilingualism specialists, it would be useful to delve deeper into the reasons behind their intermediate assessment of the importance of literacy (Table 6). Indeed, studies explain that there is a close relationship between bilingualism and literacy development, but the way in which literacy skills are acquired in a bilingual context is different from the way in which they are acquired in a monolingual context [50,51]. This fact may explain the position of bilingualism specialists, but further research is needed. It has also been shown that educators’ assessment of the importance of literacy depends strongly on the method with which they themselves learned to read (Figure 4), which is one of the original elements of this work. Thus, those who have learned with the analytical method value more, in general, the work of literacy in early childhood education, except in the case of TP specialists (Figure 4).

Regarding the impact of the pandemic and the use of facemasks, the participants believe that the pandemic has really had a negative impact on literacy learning in early childhood education (Table 5). They felt that the use of facemasks had a very negative impact in this regard. In addition, the EFA has identified that the assessments of the impact of facemasks at the visual and phonological levels are strongly correlated, which means that participants perceive that this negative influence is analogous at both levels (Table 2). All this is in line with the medical studies that have been done on the subject [36], which indicate that facemasks have impaired children’s communication in two main ways: (i) they prevent seeing the gestures of their interlocutors; and (ii) they attenuate the voice and the perceived nuances of pronunciation of sounds [52,53]. These effects of facemasks on children’s literacy development are to be expected, given the known impact that facemasks have on communication among medical professionals who use them regularly (mainly due to the decrease in visual and auditory perception that they entail) [12,54]. In this sense, there are studies that propose the use of transparent facemasks to solve, in part, the communication difficulties derived from the use of opaque masks [19,20,55]. On the other hand, other studies show that 2-year-old children can recognize familiar words when they hear them pronounced with an opaque facemask, but recognize them to a lesser extent when they are pronounced with a transparent facemask [16]. This implies that the use of transparent facemasks as a solution to the problems generated using opaque facemasks is debatable and, in any case, requires further studies. Furthermore, as far as the development of literacy in early childhood education is concerned, there are no specific studies that analyze the efficacy of the use of transparent facemasks, as far as it has been explored.

This study shows that, among Spanish educators, the perception of the use of facemasks in literacy learning depends strongly on the position of the educator in the classroom. It is noteworthy, in this sense, that bilingualism specialists are those who attribute a greater negative impact to the use of facemasks in the classroom (Table 6). This result is in line with works that demonstrate the importance of visual and phonological aspects for early second language learning [5,56,57,58]. In addition, it has been shown that the method with which the teachers surveyed learned to read conditions their perception of the influence of the facemask on literacy learning (Figure 5). Thus, those who learned with the synthetic method are those who attribute a greater negative impact in this regard. This fact is in line with other previous works that establish the existence of essential differences between the two learning methods analyzed [17], but it constitutes a novelty in the specialized literature because until now the impact of the method with which the teacher learned to read on the studied perception had not been explored. Therefore, this is an original aspect of the present study.

To comprehensively address the real affectation that the use of facemasks has on the development of literacy, it would be necessary to complete the results of the research presented here with other perspectives that address psychological and medical aspects linked to the development of children’s language. Furthermore, the study has been limited to the descriptive level. To analyze the reasons for the differences between the opinions of specialist and generalist teachers and between those who learned to read with each of the methods described (analytical or synthetic), a specific study would be necessary. On the other hand, the size of the sample used, and the distribution of the participants (mostly women) preclude a sufficiently significant quantitative study of the differences by age, experience or gender of the variables studied. Therefore, in order to study the existence of gaps in the perceptions of educators and to ensure greater representativeness of the sample, it is proposed to increase the sample size by ensuring a reasonably homogeneous distribution with respect to the sociological aspects mentioned. Finally, if the sample of professionals studied could be expanded to include teachers from different locations, it would be possible to analyze whether there is any type of geographic variable that influences the evaluations expressed.

On the other hand, it would be interesting to conduct a correlational study of teachers’ perceptions of the impact of the use of facemasks on children’s literacy learning and emotional life, in order to check whether teachers establish some kind of dependence between both areas, as the literature points out [7,18,25,30,31].

Based on the results of the study, it is suggested that teachers, specialists and generalists, work in a more coordinated manner in their respective didactic actions in the classroom. This would probably make it possible to homogenize the perceptions that the different professionals have of literacy and, therefore, the criteria for didactic action with respect to it. In addition, it would be advisable to reinforce the training in the field of literacy of pre-service teachers, both generalists and specialists.

## 5. Conclusions

Throughout the study, it was found that early childhood education professionals attribute a high importance to the learning of literacy in children at this stage and that the use of digital resources would increase the effectiveness of educational action in this regard. There is, among the respondents, the perception that the COVID-19 pandemic has slowed down the acquisition of literacy acquisition objectives. In this sense, the use of opaque facemasks is seen as a negative element for the development of the corresponding skills, both visually and phonologically. In the above perceptions, specialist teachers (TP, SH, and bilingualism specialists) value the importance of literacy learning less than generalists. However, those who attribute the greatest negative impact to the use of facemasks are the bilingualism specialists. Regarding the method (synthetic or analytical) that the participants used to learn to read, those who used the analytical method (except TP specialists) give more importance to literacy learning, but those who learned with the synthetic method identify a greater impact of the use of facemasks.

## Figures and Tables

**Table 1:** Questions of the survey.

Variable	Question Number	Question
Importance of literacy in early childhood education	1	Value the importance of the implementation of the literacy area in the early childhood education classroom
	2	Value the need for early childhood education students to begin to recognize phonemes and graphemes
	3	Value the importance of early childhood education students’ access to the next educational stage knowing how to read and write
	4	Value the degree of knowledge of literacy with which the children in your center enter primary education
Importance of the use of digital resources in the teaching of literacy	5	Value the importance of the use of digital resources for literacy learning
	6	Value the level of motivation generated in students using digital resources in the learning of literacy
Effectiveness of the analytical method	7	Value the efficacy of the analytical method as a method of teaching literacy
Effectiveness of the synthetic method	8	Value the effectiveness of the synthetic method as a method for teaching literacy
Level of impact of the pandemic on literacy	9	Value the linguistic delay that the students present when they join the school after the pandemic
	10	Compared to the pre-pandemic state, how do you value the achievement of objectives in learning to read and write?
Negative influence of the use of facemasks in the learning of literacy	11	Value the degree of negative influence that the use of the facemask has on the development of visual literacy
	12	Value the degree of negative influence that the use of the facemask has on the development of literacy at the phonological level
	13	Value the frequency with which you use support material (such as a transparent mask or microphone) to solve communication difficulties caused by the use of facemasks
Perception of the need for speech-language intervention in the classroom	14	Perception of the need for speech-language intervention in the classroom (pre-pandemic)
	15	Perception of the need for speech-language intervention in the classroom (post-pandemic)

**Table 2:** Factorial weights of the EFA.

Question	Factor 1. Importance of Literacy	Factor 2. ICT in Literacy	Factor 3. Analytical Method	Factor 4. Synthetic Method	Factor 5. Impact of Pandemic on Literacy	Factor 6. Influence of Facemasks
Q. 1	0.930					
Q. 2	0.644					
Q. 3	0.837					
Q. 4	0.942					
Q. 5		0.991				
Q. 6		0.703				
Q. 7			0.743			
Q. 8				0.769		
Q. 9					0.754	
Q. 10					0.975	
Q. 11						0.741
Q. 12						0.801
Q. 13						0.962

**Table 3:** Cumulative proportion of explained variance of the principal component analysis.

	Factor 1	Factor 2	Factor 3	Factor 4	Factor 5	Factor 6
Proportion Variance	0.108	0.132	0.103	0.093	0.116	0.091
Cumulative Variance	0.108	0.240	0.343	0.436	0.552	0.643

**Table 4:** Pearson correlation coefficients.

	Factor 1	Factor 2	Factor 3	Factor 4	Factor 5	Factor 6	Global
Factor 1	1	0.1413	0.2159	0.1995	−0.1380	0.0197	0.7190
Factor 2		1	0.1558	0.0556	0.1483	−0.0924	0.8986
Factor 3			1	0.1745	0.1172	0.1928	0.8975
Factor 4				1	0.0001	0.1401	0.8445
Factor 5					1	0.1379	0.7293
Factor 6						1	0.7196
Global							1

**Table 5:** Global mean values and standard deviations (over 5).

	Mean Values	Standard Deviations
Importance of literacy	3.31	1.42
Importance of ICT in literacy	4.12	0.84
Analytical method	3.23	1.06
Synthetic method	3.32	1.08
Impact of the pandemic on literacy	3.76	0.93
Negative influence of facemasks	4.58	0.78

**Table 6:** Mean values and standard deviations (over 5) when the participants are differentiated by their position.

Factor	Bilingualism Specialist	SH Specialist	TP Specialist	Group Tutor	Support Technician	ANOVA	*p*-Value
Importance of literacy	3.30	3.17	2.65	3.58	3.42	4.302	0.0342 *
Importance of ICT in literacy	4.20	4.33	4.00	4.50	4.08	0.8808	0.5012
Analytical method	3.00	2.50	3.38	3.67	3.25	0.8472	0.5208
Synthetic method	3.60	4.17	2.92	3.67	3.27	2.1133	0.1389
Impact of the pandemic on literacy	3.60	4.00	3.85	3.83	3.73	0.3630	0.8308
Negative influence of facemasks	4.90	4.42	4.54	4.75	4.57	4.2514	0.0363 *

* *p* < 0.05.

**Table 7:** Mean values and standard deviations (over 5) of the perception of the need for speech-language intervention in the classroom when the participants are differentiated by their position in the classroom.

Need for Speech-Language Intervention	Bilingualism Specialist	SH Specialist	TP Specialist	Group Tutor	Support Technician	ANOVA	*p*-Value
Pre-pandemic	3.40	3.50	3.85	3.16	3.50	0.3204	0.8641
Post-pandemic	4.40	4.00	4.00	3.68	4.00		

## Data Availability

The data are not publicly available because they are part of a larger project involving more researchers. If you have any questions, please ask the contact author.
